# Impact of Hypofractionated Radiotherapy on Patient-reported Outcomes in Prostate Cancer: Results up to 5 yr in the CHHiP trial (CRUK/06/016)

**DOI:** 10.1016/j.euo.2021.07.005

**Published:** 2021-12

**Authors:** John N. Staffurth, Joanne S. Haviland, Anna Wilkins, Isabel Syndikus, Vincent Khoo, David Bloomfield, Chris Parker, John Logue, Christopher Scrase, Alison Birtle, Zafar Malik, Miguel Panades, Chinnamani Eswar, John Graham, Martin Russell, Catherine Ferguson, Joe M. O’Sullivan, Clare A. Cruickshank, David Dearnaley, Emma Hall, on behalf of the CHHiP Trial Management Group

**Affiliations:** a Cardiff University/Velindre Cancer Centre, Cardiff, UK; b The Institute of Cancer Research, London, UK; c Clatterbridge Cancer Centre, Wirral, UK; d Royal Marsden NHS Foundation Trust, London, UK; e Brighton and Sussex University Hospitals, Brighton, UK; f Christie Hospital, Manchester, UK; g Ipswich Hospital, Ipswich, UK; h Rosemere Cancer Centre, Royal Preston Hospital, Preston, UK; i Lincoln County Hospital, Lincoln, UK; j Beacon Centre, Musgrove Park Hospital, Taunton, UK; k Beatson West of Scotland Cancer Centre, Glasgow, UK; l Sheffield Teaching Hospitals Foundation Trust, Sheffield, UK; m Queen’s University Belfast, Belfast, UK

**Keywords:** Prostate cancer, Hypofractionation, Patient-reported outcomes

## Abstract

**Background:**

Moderate hypofractionation is the recommended standard of care for localised prostate cancer following the results of trials including Conventional or Hypofractionated High Dose Intensity Modulated Radiotherapy in Prostate Cancer (CHHiP). Evaluation of long-term patient-reported outcomes (PROs) is important to confirm safety and enhance patient information.

**Objective:**

To determine whether 5-yr PROs from the CHHiP quality of life (QoL) substudy confirm 2-yr findings and assess patterns over follow-up.

**Design, setting, and participants:**

A phase III randomised controlled trial recruited from 2002 to 2011. The QoL substudy completed accrual in 2009; participants were followed up to 5 yr after radiotherapy. Analyses used data snapshot taken on August 26, 2016. A total of 71 radiotherapy centres were included in the study (UK, Republic of Ireland, Switzerland, and New Zealand); all 57 UK centres participated in the QoL substudy. CHHiP recruited 3216 men with localised prostate cancer (cT1b-T3aN0M0).

**Intervention:**

Conventional (74 Gy/37 fractions/7.4 wk) or hypofractionated radiotherapy (60 Gy/20 fractions/4 wk or 57 Gy/19 fractions/3.8 wk) was delivered with intensity-modulated techniques.

**Outcome measurements and statistical analysis:**

University of California Los Angeles Prostate Cancer Index, Short Form 36 and Functional Assessment of Cancer Therapy—Prostate, or Expanded Prostate Cancer Index Composite and Short Form 12 questionnaires were administered at baseline, before radiotherapy, at 10 wk, and at 6, 12, 18, 24, 36, 48, and 60 mo after radiotherapy. The QoL primary endpoint was overall bowel bother.

**Results and limitations:**

The QoL substudy recruited 2100 patients; 1141 5-yr forms were available from 1957 patients still alive (58%). There were no statistically significant differences in 5-yr prevalence of overall “moderate or big” bowel bother: 19/349 (5.4%), 29/381 (7.6%), and 21/393 (5.3%) for 74, 60, and 57 Gy, respectively; overall urinary or sexual bother at 5 yr was similar between schedules. Bowel and urinary symptoms remained stable from 2 to 5 yr for all schedules. Some evidence of worsening overall sexual bother from baseline to 5 yr was less likely in the hypofractionated schedules compared with 74 Gy (odds ratios for increase in bother score vs 74 Gy: 0.55 [0.30–0.99], *p* = 0.009 for 60 Gy, and 0.52 [0.29–0.94], *p* = 0.004 for 57 Gy). General QoL scores were similar between schedules at 5 yr.

**Conclusions:**

Longer follow-up confirms earlier findings, with similar patient-reported bowel, urinary, and sexual problems between schedules overall. The continued low incidence of moderate or high bother confirms that moderate hypofractionation should be the standard of care for intermediate-risk localised prostate cancer.

**Patient summary:**

We looked at patient-reported outcomes up to 5 yr after treatment in a trial of different radiotherapy schedules for prostate cancer. The findings confirmed that shorter radiotherapy schedules were as safe as standard radiotherapy in terms of bowel, urinary, and sexual problems.

**Take  Home Message:**

Bowel, urinary, and sexual symptoms were similar between schedules up to 5 yr. The continued low incidence of moderate/high bother confirms that moderate hypofractionated radiotherapy should be considered the standard of care for men with intermediate-risk prostate cancer.

## 1 Introduction

Prostate cancer remains the most common cancer in men in the UK [1]. External beam radiotherapy, radical prostatectomy, and brachytherapy are standard options for radical treatment for localised disease, considered to have equivalent tumour control at least up to 10 yr [2]. Patients and physicians balance efficacy against side effects in decision-making [3]. Patient-reported outcomes (PROs) detect treatment side effects more reliably than clinical assessments [4], [5].

The Conventional or Hypofractionated High Dose Intensity Modulated Radiotherapy in Prostate Cancer (CHHiP) trial randomised 3216 men with localised prostate cancer undergoing radiotherapy to conventional fractionation (74 Gy in 37 fractions) versus one of two moderately hypofractionated regimens (60 Gy in 20 fractions and 57 Gy in 19 fractions). At 5.2-yr median follow-up, the 60 Gy schedule was shown to be noninferior to conventional fractionation with 5-yr biochemical or clinical failure–free rates of 90.6% (95% confidence interval 88.5–92.3) and 88.3% (86.0–90.2), respectively; the 57 Gy schedule was not noninferior (85.9%, 83.4–88.0) [6]. Five-year clinician-reported late genitourinary (GU) and gastrointestinal (GI) toxicity was similar between schedules [6]. These results, with those from other fractionation trials [7], [8], have led to the recommendation of moderate hypofractionation as the standard of care for external beam radiotherapy [9], [10], [11].

Published results from the CHHiP quality of life (QoL) substudy up to 2-yr follow-up showed similar incidence of patient-reported bowel and urinary symptoms between schedules [12]; we report results to 5 yr, as there is evidence of increasing cumulative incidence of late effects beyond the 2-yr time period for both conventional and hypofractionated radiotherapy [13], [14].

## 2 Patients and methods

### 2.1 Study design and participants

The CHHiP trial included 3216 men recruited from 71 centres from September 2002 to June 2011; full details of design, eligibility, and treatment have been published [15]. Participation in the QoL substudy was open to all 57 UK centres, and 2100 patients were accrued by November 2009. The CHHiP trial is registered (ISRCTN97182923).

### 2.2 Procedures

Men were registered before or after starting hormone therapy. Patients with National Comprehensive Cancer Network (NCCN) intermediate- or high-risk disease received short-course androgen suppression for 3–6 mo before and during radiotherapy (optional for those with low-risk disease). Participants consenting to the QoL substudy were eligible to complete questionnaires at trial entry if they had not commenced hormonal therapy, to minimise the impact of hormones. Questionnaires were administered before radiotherapy, at 10 wk, and at 6, 12, 18, 24, 36, 48, and 60 mo after the start of radiotherapy. Full details of QoL instruments have been published [12], [15]. Between 2002 and early 2009, QoL measures consisted of the University of California Los Angeles Prostate Cancer Index (UCLA-PCI) [16], including the Short Form 36 (SF-36) and Functional Assessment of Cancer Therapy-Prostate (FACT-P) [17]. Following a protocol amendment in 2009, the Expanded Prostate Cancer Index Composite (EPIC) [18] and Short Form 12 (SF-12) QoL instruments were used for newly randomised patients, as EPIC was emerging as the international standard QoL instrument for men having radiotherapy [19]. EPIC-50 assessed bowel and urinary domains and EPIC-26 sexual and hormonal domains [20].

For all QoL instruments, a higher score represents better QoL. All questionnaires were scored according to the recommended manuals.

The primary QoL endpoint was overall bowel bother, reported on a five-point scale (none, very small, small, moderate, and big bother) from EPIC or UCLA-PCI. Key secondary endpoints were overall urinary bother and overall sexual bother. Other secondary endpoints were related to individual bowel, urinary, and sexual items and domain scores from EPIC and UCLA-PCI, and general health-related quality of life (HRQoL) domains from FACT-P, SF-36, and SF-12.

### 2.3 Statistical analysis

Each pair of schedules was compared, with statistical tests at 5 yr. CHHiP was not originally powered for QoL analyses; retrospective calculations were reported previously [12].

**Fig. 1:**
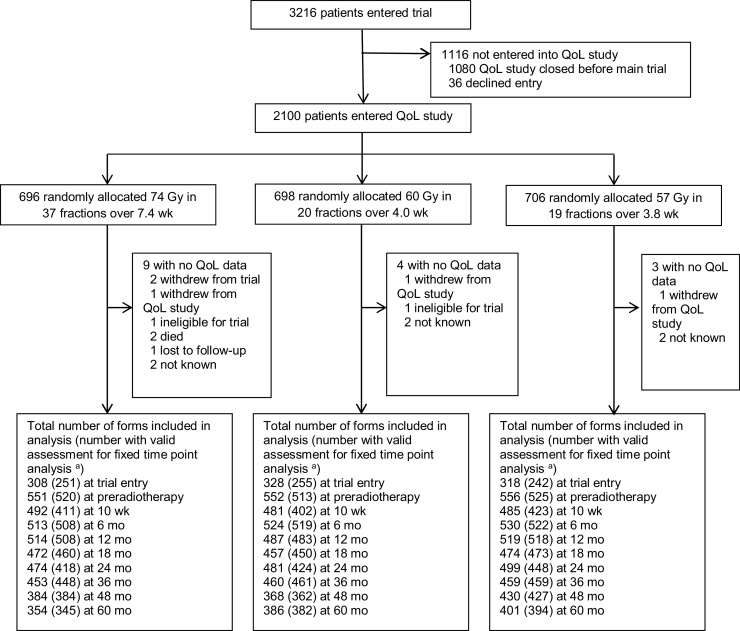
CONSORT diagram. QoL = quality of life. ^a^ Patients were excluded from the fixed time-point analyses if their QoL assessments were dated outside prespecified acceptable time intervals: after 1 mo of endocrine therapy or after randomisation for baseline, before 3 mo or after 1 wk of starting radiotherapy for preradiotherapy, outside 2 wk from the expected date of completion for 10 wk, outside 3 mo from the expected date of completion for 6–24 mo, and outside 6 mo for 36–60 mo.

Cross-sectional analyses compared groups at 5 yr using the Mantel-Haenszel chi-square trend test and the Mann-Whitney test. Moderate and severe events were combined due to few severe events. Patients were excluded from cross-sectional analyses if their QoL assessments were outside prespecified time intervals (Fig. 1).

Change from baseline (calculated as postradiotherapy score minus baseline score) was assessed to account for differences in pre-existing comorbidity between groups. As more questionnaires were available at the preradiotherapy time point than at the prehormone time point (baseline), preradiotherapy data were used as a surrogate baseline for bowel and urinary symptoms unless missing, in which case baseline data were used. For sexual endpoints and general HRQoL, prehormone data were used as baseline, as endocrine treatment had a marked influence on these scores at the preradiotherapy time point; patients with no prehormone therapy data were excluded from analyses requiring baseline data. Change from baseline was also presented in terms of proportion of patients experiencing a minimally important difference according to published thresholds [21], [22].

For the individual items of overall bowel, urinary, and sexual bother, the odds of change in score from baseline to 5 yr were modelled using ordinal logistic regression [23] and schedules compared using odds ratios (ORs, with 99% confidence interval [CI]), where OR < 1 favour the hypofractionated schedules, indicating lower odds of an increase in bother score than in the 74 Gy group. Analysis of covariance modelling was used to assess change from baseline to 5 yr for continuous variables such as domain scores, adjusting for baseline score.

Time to emergent “small or worse” or “moderate or worse” toxicity from 6 mo was assessed for individual endpoints using survival analysis methods, excluding patients who had already experienced an event at trial entry or before radiotherapy. Endpoints were defined as for the 2-yr analyses, censoring patients at the date of last QoL assessment or date of death, whichever occurred sooner. Kaplan-Meier cumulative incidence rates of emergent toxicity were estimated (with 99% CI), and schedules were compared using the log rank test. Hazard ratios (HRs; with 99% CI) were obtained from Cox proportional hazard regression.

There was no imputation of missing questionnaires; domain scores were calculated only if sufficient items were completed according to the relevant scoring manual. Guidance for the EPIC measure specifies that domain scores can be calculated if >80% of the items within a scale are completed [24]; for UCLA-PCI, the rule is that >50% of items should be completed within a scale [25].

All hypotheses for the PRO endpoints were two sided. There was no formal adjustment of *p* values to allow for multiple testing, but the statistical analysis plan prespecified a conservative cutoff of 0.001 to indicate statistical significance due to the large number of endpoints and hypotheses tested; similarly, 99% CI was used.

The analysis was carried out on an intention to treat basis, using STATA v13.1.

## 3 Results

### 3.1 Patients

Baseline characteristics of the 2100 men in the CHHiP QoL substudy have been reported [12]. Questionnaire return rates from patients remaining eligible (alive and not withdrawn) were over 90% at all time points up to 2 yr, then 88%, 75%, and 74% at 3, 4, and 5 yr, respectively (Fig. 1, CONSORT diagram). Baseline characteristics were similar for patients with and without 5-yr QoL data, except for patients with higher T stage and higher NCCN risk group being less likely to return a 5-yr questionnaire (Supplementary Table 1). By 5 yr, 143 patients had died (57 in the 74 Gy, 36 in the 60 Gy, and 50 in the 57 Gy group).

### 3.2 Bowel, urinary, and sexual problems

#### 3.2.1 Prevalence of symptoms

Five-year prevalence of small or worse overall bowel bother was 52/349 (14.9%) for 74 Gy, 58/381 (15.2%) for 60 Gy, and 61/393 (15.5%) for 57 Gy; corresponding figures for moderate or worse bowel bother were 19/349 (5.4%), 29/381 (7.6%), and 21/393 (5.3%), respectively (Table 1). Five-year prevalence of overall urinary bother was 58/341 (17.0%), 63/377 (16.7%), and 62/382 (16.2%) with small or worse symptoms, and 23/341 (6.7%), 35/377 (9.3%), and 30/382 (7.8%) with moderate or worse symptoms, for 74, 60, and 57 Gy, respectively. Five-year prevalence of small or worse overall sexual bother was 192/333 (57.7%) for 74 Gy, 187/363 (51.5%) for 60 Gy, and 198/376 (52.7%) for 57 Gy; the prevalence of moderate or worse sexual bother was 139/333 (41.7%), 133/363 (36.6%), and 153/376 (40.7%), respectively (Table 2).

**Table 1:** Individual bowel symptoms at 5 yr for UCLA-PCI and EPIC QoL instruments

Bowel symptoms	5 yr			60 vs 74 Gy	57 vs 74 Gy	60 vs 57 Gy
	74 Gy/37 f	60 Gy/20 f	57 Gy/19 f	*p* value a	*p* value a	*p* value a
	*N* (%)	*N* (%)	*N* (%)			
Overall bowel bother (problem); UCLA-PCI and EPIC	*N* = 349	*N* = 381	*N* = 393	0.81	0.69	0.52
None	210 (60.2)	237 (62.2)	256 (65.1)			
Very small	87 (24.9)	86 (22.6)	76 (19.3)			
Small	33 (9.5)	29 (7.6)	40 (10.2)			
Moderate	16 (4.6)	21 (5.5)	11 (2.8)			
Big	3 (0.9)	8 (2.1)	10 (2.5)			
Rectal urgency (problem); UCLA-PCI and EPIC	*N* = 341	*N* = 375	*N* = 383	0.56	0.70	0.83
None	246 (72.1)	293 (78.1)	286 (74.7)			
Very small	51 (15.0)	28 (7.5)	49 (12.8)			
Small	14 (4.1)	19 (5.1)	15 (3.9)			
Moderate	20 (5.9)	26 (6.9)	21 (5.5)			
Big	10 (2.9)	9 (2.4)	12 (3.1)			
Faecal incontinence (problem); EPIC	*N* = 82	*N* = 96	*N* = 90	0.30	0.86	0.33
None	75 (91.5)	83 (86.5)	79 (87.8)			
Very small	5 (6.1)	9 (9.4)	10 (11.1)			
Small	1 (1.2)	2 (2.1)	1 (1.1)			
Moderate	1 (1.2)	1 (1.0)	0 (0)			
Big	0 (0)	1 (1.0)	0 (0)			
Rectal bleeding (problem); EPIC	*N* = 82	*N* = 96	*N* = 90	0.22	0.70	0.44
None	73 (89.0)	80 (83.3)	81 (90.0)			
Very small	8 (9.8)	13 (13.5)	7 (7.8)			
Small	0 (0)	0 (0)	0 (0)			
Moderate	1 (1.2)	2 (2.1)	0 (0)			
Big	0 (0)	1 (1.0)	2 (2.2)			
Loose or liquid stools (problem); UCLA-PCI and EPIC	*N* = 340	*N* = 377	*N* = 382	0.67	0.30	0.54
None	150 (44.1)	157 (41.6)	145 (38.0)			
Very small	135 (39.7)	158 (41.9)	174 (45.5)			
Small	38 (11.2)	42 (11.1)	42 (11.0)			
Moderate	11 (3.2)	14 (3.7)	15 (3.9)			
Big	6 (1.8)	6 (1.6)	6 (1.6)			
Frequency of bowel movements/d; EPIC	*N* = 83	*N* = 96	*N* = 94	0.76	0.50	0.31
<3	72 (86.7)	82 (85.4)	84 (89.4)			
3–4	9 (10.8)	11 (11.5)	9 (9.6)			
5+	2 (2.4)	3 (3.1)	1 (1.1)			
Crampy pain in abdomen/pelvis (problem); UCLA-PCI and EPIC	*N* = 345	*N* = 380	*N* = 393	0.85	0.79	0.64
None	302 (87.5)	331 (87.1)	341 (86.8)			
Very small	23 (6.7)	25 (6.6)	28 (7.1)			
Small	9 (2.6)	12 (3.2)	16 (4.1)			
Moderate	5 (1.4)	5 (1.3)	6 (1.5)			
Big	6 (1.7)	7 (1.8)	2 (0.5)			
Bowel distress; UCLA-PCI	*N* = 258	*N* = 281	*N* = 294	0.74	0.93	0.80
None	189 (73.3)	206 (73.3)	215 (73.1)			
Small	53 (20.5)	60 (21.4)	61 (20.7)			
Moderate	12 (4.7)	13 (4.6)	15 (5.1)			
Severe	4 (1.6)	2 (0.7)	3 (1.0)			

EPIC = Expanded Prostate Cancer Index Composite; f = fractions; QoL = quality of life; UCLA-PCI = University of California Los Angeles Prostate Cancer Index.

a *p* value from χ^2^ trend test.

**Table 2:** Individual urinary and sexual symptoms at 5 yr for UCLA-PCI and EPIC QoL instruments

Endpoints	5 yr			60 vs 74 Gy	57 vs 74 Gy	60 vs 57 Gy
	74 Gy/37 f	60 Gy/20 f	57 Gy/19 f	*p* value a	*p* value a	*p* value a
	*N* (%)	*N* (%)	*N* (%)			
*Urinary symptoms*						
Overall urinary bother (problem); UCLA-PCI and EPIC	*N* = 341	*N* = 377	*N* = 382	0.99	0.68	0.68
None	190 (55.7)	225 (59.7)	229 (59.9)			
Very small	93 (27.3)	89 (23.6)	91 (23.8)			
Small	35 (10.3)	28 (7.4)	32 (8.4)			
Moderate	20 (5.9)	25 (6.6)	23 (6.0)			
Big	3 (0.9)	10 (2.7)	7 (1.8)			
Urinary control; UCLA-PCI and EPIC	*N* = 343	*N* = 378	*N* = 388	0.27	0.58	0.59
Total control	199 (58.0)	243 (64.3)	243 (62.6)			
Occasional dribbling	134 (39.1)	122 (32.3)	127 (32.7)			
Frequent dribbling	10 (2.9)	9 (2.4)	16 (4.1)			
No control	0 (0)	4 (1.1)	2 (0.5)			
Use of urinary pads/day; UCLA-PCI and EPIC	*N* = 339	*N* = 374	*N* = 380	>0.99	0.74	0.73
None	245 (72.3)	266 (71.1)	279 (73.4)			
1–2	93 (27.4)	106 (28.3)	99 (26.1)			
3+	1 (0.3)	2 (0.5)	2 (0.5)			
Haematuria (problem); EPIC	*N* = 83	*N* = 97	*N* = 96	0.68	0.22	0.23
None	82 (98.8)	95 (97.9)	92 (95.8)			
Very small	0 (0)	1 (1.0)	1 (1.0)			
Small	1 (1.2)	0 (0)	1 (1.0)			
Moderate	0 (0)	1 (1.0)	1 (1.0)			
Big	0 (0)	0 (0)	1 (1.0)			
Dysuria (problem); EPIC	*N* = 83	*N* = 97	*N* = 96	0.16	0.05	0.48
None	81 (97.6)	92 (94.8)	88 (91.7)			
Very small	2 (2.4)	2 (2.1)	4 (4.2)			
Small	0 (0)	1 (1.0)	0 (0)			
Moderate	0 (0)	1 (1.0)	3 (3.1)			
Big	0 (0)	1 (1.0)	1 (1.0)			
*Sexual symptoms*						
Overall sexual bother (problem); UCLA-PCI and EPIC	*N* = 333	*N* = 363	*N* = 376	0.05	0.15	0.64
None	83 (24.9)	112 (30.9)	128 (34.0)			
Very small	58 (17.4)	64 (17.6)	50 (13.3)			
Small	53 (15.9)	54 (14.9)	45 (12.0)			
Moderate	45 (13.5)	51 (14.0)	55 (14.6)			
Big	94 (28.2)	82 (22.6)	98 (26.1)			
Erection quality (problem); UCLA-PCI and EPIC	*N* = 333	*N* = 363	*N* = 379	0.19	0.18	0.98
None	60 (18.0)	85 (23.4)	82 (21.6)			
Small	77 (23.1)	79 (21.8)	89 (23.5)			
Moderate	80 (24.0)	80 (22.0)	90 (23.7)			
Severe	116 (34.8)	119 (32.8)	118 (31.1)			
Erection frequency (problem); UCLA-PCI and EPIC	*N* = 332	*N* = 363	*N* = 376	0.06	0.08	0.87
None	29 (8.7)	41 (11.3)	40 (10.6)			
Very small	30 (9.0)	40 (11.0)	53 (14.1)			
Small	38 (11.4)	55 (15.2)	37 (9.8)			
Moderate	51 (15.4)	44 (12.1)	54 (14.4)			
Big	184 (55.4)	183 (50.4)	192 (51.1)			
Woken with erection morning/night (problem); UCLA-PCI	*N* = 259	*N* = 279	*N* = 294	0.02	0.04	0.66
None	1 (0.4)	11 (3.9)	4 (1.4)			
Very small	14 (5.4)	25 (9.0)	24 (8.2)			
Small	33 (12.7)	36 (12.9)	38 (12.9)			
Moderate	73 (28.2)	66 (23.7)	95 (32.3)			
Big	138 (53.3)	141 (50.5)	133 (45.2)			

EPIC = Expanded Prostate Cancer Index Composite; f = fractions; QoL = quality of life; UCLA-PCI = University of California Los Angeles Prostate Cancer Index.

a *p* value from χ^2^ trend test.

**Fig. 2:**
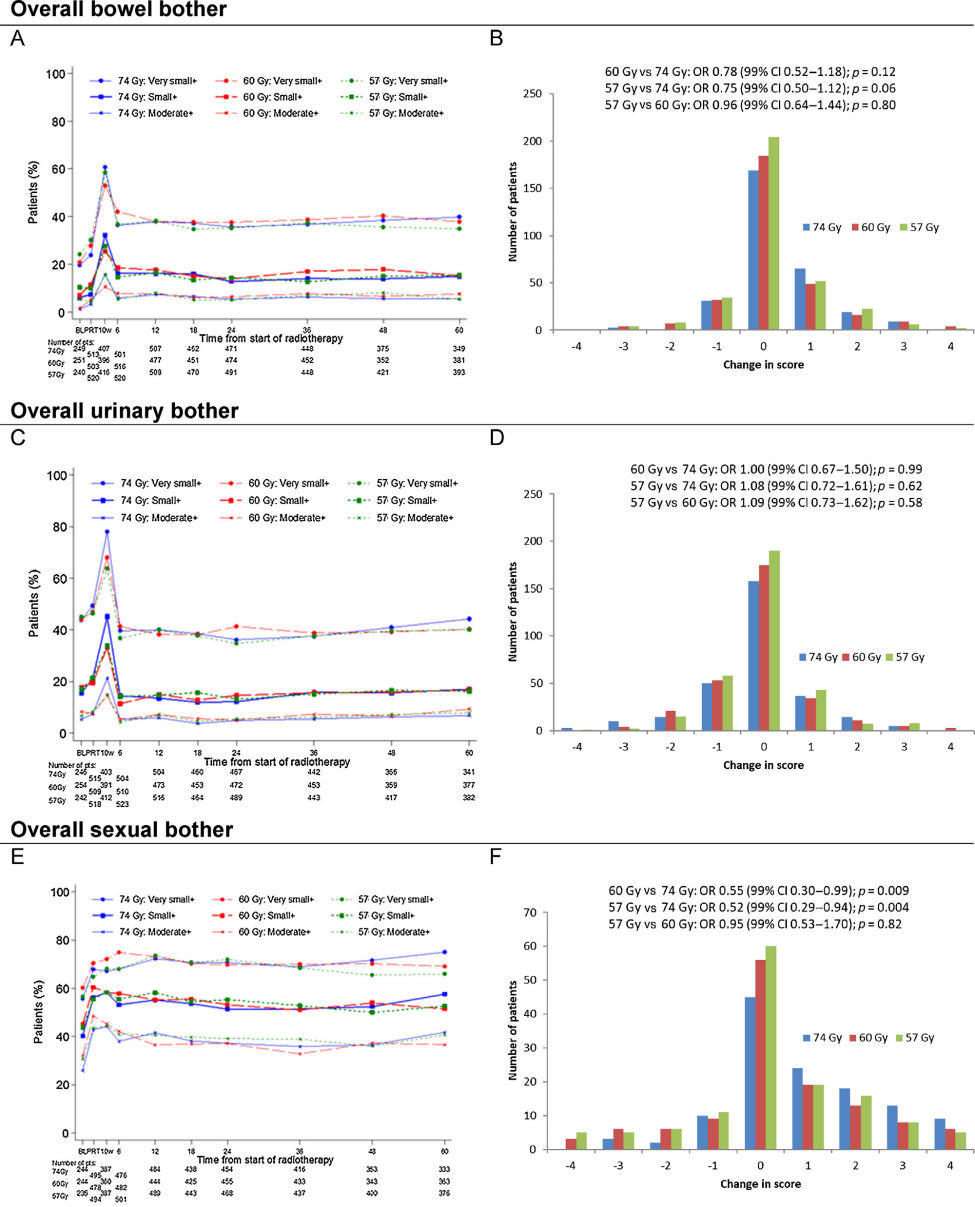
Overall bowel, urinary, and sexual bother. Data are (A) prevalence of overall bowel bother, (B) change from preradiotherapy time point to 5 yr for overall bowel bother, (C) prevalence of overall urinary bother, (D) change from preradiotherapy time point to 5 yr for overall urinary bother, (E) prevalence of overall sexual bother, and (F) change from preradiotherapy time point to 5 yr for overall sexual bother. A negative change in bother score from baseline/before RT to 5 yr indicates an improvement in QoL; a positive change in bother score represents worsening QoL. Odds ratios <1 favour the hypofractionated schedules, indicating lower odds of an increase in bother score than in the 74 Gy group. CI = confidence interval; OR = odds ratio; pts = patients; QoL = quality of life; RT = radiotherapy.

Frequencies of overall bowel, urinary, and sexual bother were similar between schedules at all time points (Fig. 2). There were no statistically significant differences between the schedules at 5 yr for any of the individual bowel, urinary, or sexual symptoms or the corresponding domain scores (Table 1, Table 2, and Supplementary Table 2). At 5 yr, the most common bowel symptom was loose or liquid stools, with small or worse problems reported by 55/340 (16.2%) in the 74 Gy, 62/377 (16.4%) in the 60 Gy, and 63/382 (16.5%) in the 57 Gy group (Table 1). The most common urinary problem at 5 yr was lack of urinary control, with loss of control reported at least occasionally by 144/343 (42.0%) in the 74 Gy, 135/378 (35.7%) in the 60 Gy, and 145/388 (37.4%) in the 57 Gy group (Table 2). Over follow-up, the prevalence and severity of sexual problems were higher than those of bowel and urinary symptoms (Fig. 2, Table 1, Table 2, and Supplementary Table 2). At 5 yr, 244/259 (94.2%) of the 74 Gy, 243/279 (87.1%) of the 60 Gy, and 266/294 (90.5%) of the 57 Gy group reported small or worse problems with being awoken with an erection in the morning or at night, with around half of the patients rating this as a big problem (Table 2). In contrast, the majority of bowel and urinary symptoms were reported as small or moderate problems.

#### 3.2.2 Change over time

**Fig. 3:**
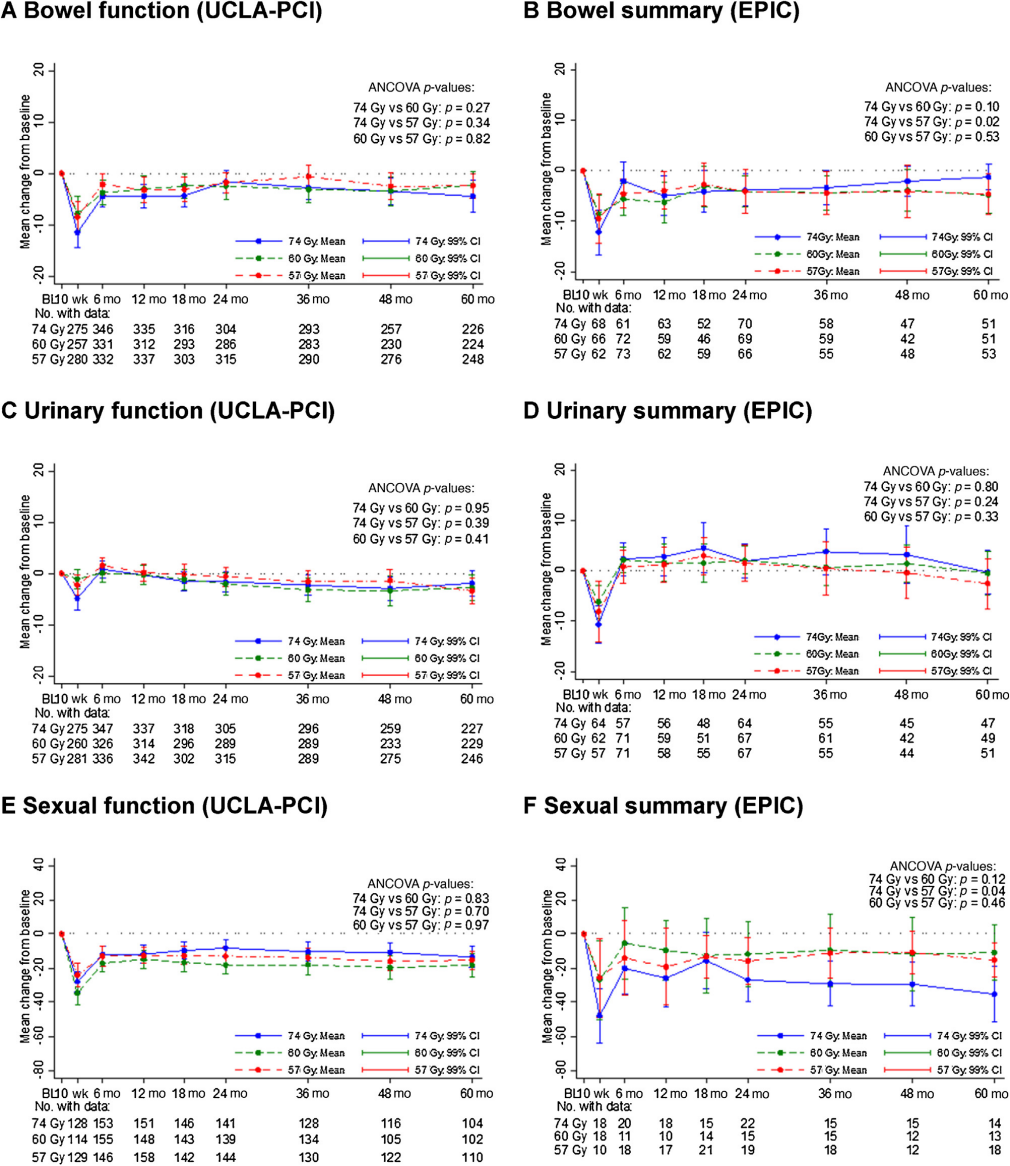
Change in bowel, urinary, and sexual domain scores from baseline up to 5 yr: (A) bowel function (UCLA-PCI), (B) bowel summary (EPIC), (C) urinary function (UCLA-PCI), (D) urinary summary (EPIC), (E) sexual function (UCLA-PCI), and (F) sexual summary (EPIC). Data shown are mean and 99% CI. Change in domain score was calculated as postradiotherapy score minus baseline score; a negative change from baseline to 5 yr indicates worsening QoL. ANCOVA = analysis of covariance; CI = confidence interval; EPIC = Expanded Prostate Cancer Index Composite; QoL = quality of life; UCLA-PCI = University of California Los Angeles Prostate Cancer Index.

Following temporary worsening in overall bowel and urinary bother at 10 wk after radiotherapy, the prevalence of overall bowel, urinary, and sexual bother changed little between 6 mo and 5 yr (Fig. 2A, 2C, and 2E). Overall, from baseline to the 5-yr time point, 558/937 patients (59.6%) had no change in overall bowel bother, 523/921 (56.8%) had no change in overall urinary bother, and 161/385 (41.8%) had no change in sexual bother (Fig. 2B, 2D, and 2F). Patterns of change in overall bowel and urinary bother scores from baseline to 5 yr were similar between the schedules (Fig. 2B and 2D). There was some evidence of an increase in sexual bother from baseline to 5 yr for 74 Gy but not the hypofractionated schedules (OR for increase in sexual bother score for 60 vs 74 Gy: 0.55 [0.30–0.99], *p* = 0.009, and for 57 vs 74 Gy: 0.52 [0.29–0.94], *p* = 0.004; Fig. 2F). An assessment of the mean change in domain scores from baseline to each time point indicated that bowel, urinary, and sexual functions were stable from 6 mo to 5 yr following radiotherapy, with sexual functioning showing the greatest decline at 5 yr compared with baseline (Fig. 3A, 3C, and 3E). Bowel and urinary summary domain scores showed only marginally worse symptoms at 5 yr than at baseline, but a greater decline for the sexual summary score was observed for all groups, particularly for 74 Gy, at 5 yr (Fig. 3B, 3D, and 3F). Changes from baseline to 5 yr for bowel and urinary function and summary scores were less than previously reported minimal important differences (MIDs) for the majority of patients, but not so for the sexual function and summary scores, although denominators were smaller for sexual domain scores (Supplementary Table 3). There were no statistically significant differences between schedules in bowel, urinary, and sexual domain scores at 5 yr adjusting for baseline (Fig. 3).

#### 3.2.3 Time to event analyses

There were no statistically significant differences between schedules for time to small or worse, and moderate or worse bowel, urinary, and sexual problems (Supplementary Tables 4 and 5, and Supplementary Fig. 1). HRs were very similar to those reported in the 2-yr analyses [12]. There was some evidence of higher rates of moderate or worse faecal incontinence in the hypofractionated schedules compared with 74 Gy (HRs vs 74 Gy: 5.75 [1.15–28.88], *p* = 0.002 for 60 Gy, and 4.17 [0.80–21.70], *p* = 0.015 for 57 Gy; Supplementary Table 4). For all treatment groups together, 5-yr cumulative incidence of overall bowel bother was 38.0% for small or worse symptoms (99% CI 33.5–42.9) and 19.5% for moderate or worse symptoms (16.4–23.2); the corresponding figures of overall urinary bother were 30.9% for small or worse symptoms (26.5–35.8) and 17.8% for moderate or worse symptoms (14.4–21.8), and those of overall sexual bother were 69.8% for small or worse symptoms (64.1–75.3) and 55.1% for moderate or worse symptoms (49.7–60.7).

### 3.3 General HRQoL domains

**Fig. 4:**
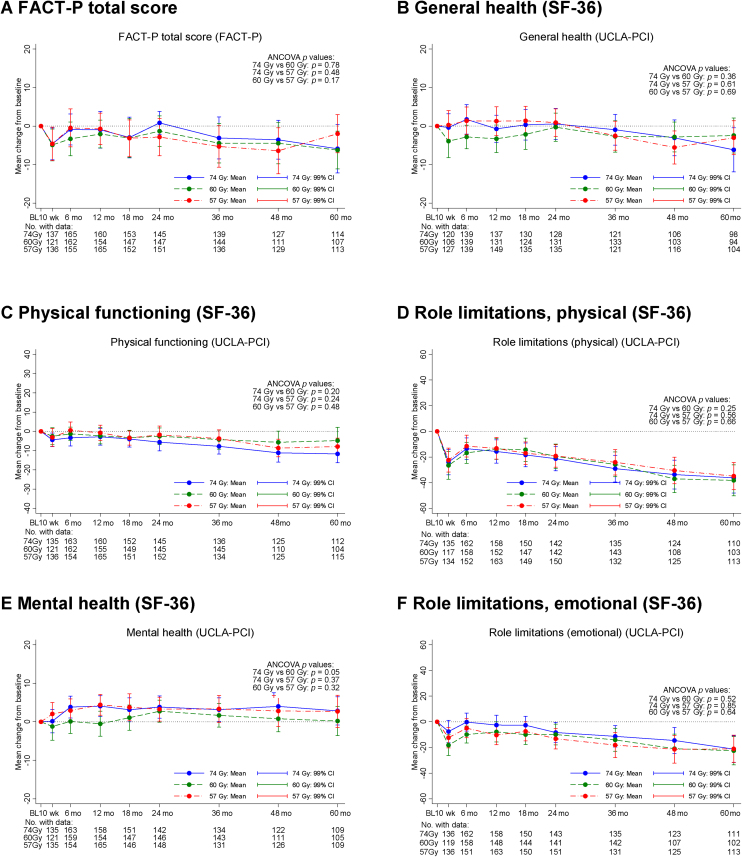
Change in general HRQoL domain scores from baseline up to 5 yr: (A) FACT-P total score, (B) general health (SF-36), (C) physical functioning (SF-36), (D) role limitations—physical (SF-36), (E) mental health (SF-36), and (F) role limitations—emotional (SF-36). Data shown are mean and 99% CI. Change in domain score was calculated as postradiotherapy score minus baseline score; a negative change from baseline to 5 yr indicates worsening QoL. ANCOVA = analysis of covariance; CI = confidence interval; FACT-P = Functional Assessment of Cancer Therapy—Prostate; HRQoL = health-related QoL; QoL = quality of life; SF-36 = Short Form 36.

At 5 yr, HRQoL domains indicating poorest QoL were role limitations (physical) and vitality from the SF-36 (Supplementary Table 6). HRQoL domain scores were similar between schedules at 5 yr (Supplementary Table 6) and when adjusting for baseline score (Fig. 4). An assessment of the mean change in scores from baseline to each time point indicated that whilst some HRQoL domains were stable from 2 to 5 yr, others such as role limitations (physical and emotional) declined (Fig. 4). Changes from baseline to 5 yr for most general HRQoL domains (except for role limitations—physical) were less than previously reported MIDs for the majority of patients (Supplementary Table 7).

## 4 Discussion

In general, PROs from the CHHiP trial up to 5 yr following radiotherapy showed a similar prevalence of overall bowel, urinary, and sexual problems between fractionation schedules. Five-year rates of bowel and urinary bother were low, but sexual problems remained prevalent, consistent with 2-yr findings [12]. Bowel and urinary problems changed little from 6 mo to 5 yr, with some evidence of worsening in sexual problems (using EPIC), especially in the 74 Gy group. Five-year estimates of relative differences between schedules from time to event analyses did not show any statistically significant differences using our predefined significance level of *p* = 0.001, consistent with our earlier results [12]. There was a larger decline from baseline in EPIC Bowel summary scores for the moderately hypofractionated schedules compared with conventional fractionation. This was driven by low-grade faecal incontinence, which is not assessed in UCLA-PCI, supporting our decision to switch questionnaires, and was not reflected in results for overall bowel bother. There was a markedly lower decline from baseline in EPIC Sexual summary scores (but not UCLA-PCI) for the moderately hypofractionated schedules compared with conventional fractionation. General HRQoL domains showed declines in role limitations (physical) and vitality functioning, but no statistically significant differences between schedules.

Our results are interesting when compared with the PRO results of the Prostate Testing for Cancer and Treatment (ProtecT) trial, in which 1643 men with screen-detected low- or intermediate-risk prostate cancer were randomised to active monitoring, radical prostatectomy, or radiotherapy to the same dose of 74 Gy in 37 fractions with neoadjuvant androgen deprivation [26]. ProtecT reported PROs on four domains (urinary, sexual, and bowel function using EPIC, and HRQoL) at baseline at the time of diagnosis, at 6 and 12 mo after randomisation, and annually thereafter. The changes in PROs over time in the radiotherapy arm of ProtecT were remarkably consistent with our results from all arms in CHHiP; they reported that bowel, urinary, and sexual function deteriorated at 6 mo but had recovered towards baseline by 12 mo, remaining stable until 6 yr of follow-up. Bowel symptoms in the radiotherapy group showed a small long-term difference from baseline (a mean reduction in bowel summary score of –3.8 at 5 yr) unlike the other groups; urinary and sexual outcomes became similar to, and often better than, the active monitoring group, which showed a steady decline over time. The mean EPIC urinary summary score fell in the radical prostatectomy arm from 91.9 at baseline to 80.1 at 6 mo driven by urinary incontinence, although there was partial recovery by 12 mo (mean score 88.1), which was maintained for long-term follow-up. Erectile firmness deteriorated in the radiotherapy and active monitoring groups with time, suggesting that some of the changes seen in our study may be the effects of ageing. CHHiP analyses suggest that refining radiotherapy dose to penile bulb and rectum will improve sexual function and reduce further rectal side effects [27], [28]. A population-based cohort study comparing treatment options in 1386 men with favourable-risk and 619 with unfavourable-risk prostate cancer showed similar patterns to our findings regarding changes in patient-reported urinary, bowel, and sexual outcomes up to 5 yr following external beam radiotherapy [29]. Taken together, these results are highly encouraging that there is no major change in the relative risks of late side effects between conventional and hypofractionated radiotherapy between 2 and 5 yr after radiotherapy.

NRG Oncology 0415 randomised men with low-risk disease between 73.8 Gy in 41 fractions (conventional) versus 70 Gy in 28 fractions (hypofractionated; *N* = 1115) [30] and reported similar scores between schedules for EPIC domains, anxiety, depression, and generic HRQoL at 5.8-yr median follow-up, consistent with our results. They enumerated changes from baseline for mean EPIC bowel, urinary, and sexual scores from baseline to 12 mo as –3.7, –0.3 and –8.2 (bowel, urinary, sexual—conventional) and –7.5, –1.8, and –8.4 (hypofractionated), respectively. Except for the difference in change in sexual function that we presume to be due to the use of hormonal therapy in the CHHiP study, these are consistent with our results for changes from baseline to 12 mo for EPIC scores of –5.0, 2.8, and –26.0 (bowel, urinary, sexual—74 Gy); –6.3, 1.6, and –9.6 (bowel, urinary, sexual—60 Gy); and –3.9, 1.2, and –19.3 (bowel, urinary, sexual—57 Gy). HYPRO randomised 820 intermediate- or high-risk prostate cancer patients to 64.6 Gy in 19 fractions or 78 Gy in 39 fractions; rates of GI or GU symptoms increased in the first 6–12 mo and then remained stable up to 5 yr [31]. In contrast to our findings, sexual activity showed continued improvement at 5 yr towards baseline levels following a dip at 6 mo; there was some recovery of sexual function in the hypofractionated group. After 3 yr, the incidence of clinically relevant deterioration of urinary symptoms was 33% for both schedules, with GI symptom decline in 38% and 36% for the hypofractionated and conventional schedules, respectively; hence, noninferiority of hypofractionation was not demonstrated for these patient-reported symptoms.

Shaikh et al [32] reported PROs from 303 men randomised to 76 Gy in 38 fractions or 70.2 Gy in 26 fractions; patients with high-risk disease had elective pelvic nodal irradiation. Overall, no differences between the schedules were seen in any domain, although lower EPIC urinary incontinence scores were reported in the hypofractionated schedule with longer-term follow-up. HRQoL outcomes were generally stable over time.

Widmark et al [33] reported the Scandinavian phase III HYPO-RT-PC trial of 1200 men with mainly intermediate-risk prostate cancer randomised to 78 Gy in 39 daily fractions versus 42.7 Gy in seven fractions (ultrafractionation) delivered on alternate days without androgen suppression. Frequencies of GI, GU, or sexual symptoms 5 yr after radiotherapy were similar between groups, although patients reported higher levels of acute toxicity and urinary symptoms at 12 mo following ultrafractionation. Rates of GU and GI symptoms were stable after 6 mo, but sexual function deteriorated with time. Fransson et al [34] have reported their QoL data 6 yr after treatment using the Prostate Cancer Symptom Scale and European Organization for Research and Treatment of Cancer Quality-of-Life Questionnaire (EORTC QLQ-C30). Although a higher proportion of men in the ultrafractionation arm had clinically relevant deterioration in bowel symptoms at the end of radiotherapy, there were no clinically relevant differences in later time points for urinary, bowel, or sexual functioning between the arms. At the 6-yr follow-up, the incidence of clinically relevant deterioration between the groups for both overall urinary and bowel bother was 33% for conventional fractionation and 28% for ultrahypofractionation, and for overall sexual bother these were 60% and 50%, respectively.

Findings show that the long-term tolerance of the CHHiP technique is excellent and that urinary and bowel PROs are stable from 6 mo after treatment. Furthermore, treatment techniques have evolved significantly since the CHHiP trial; only 30% of 900 CHHiP patients with data available were treated with daily online image-guided radiotherapy (IGRT). Fractionation guidelines from the UK Royal College of Radiologists updated in 2019 recommend that intensity-modulated radiotherapy or arc techniques including volumetric arc therapy are used [35]. These would be expected to reduce the incidence of late GU, GI, and sexual effects; further benefits may occur with other advances such as IGRT [36] or implanted hydrogel spacers [37].

Strengths of the CHHiP QoL substudy include the large sample size and questionnaire return rates. Although questionnaire returns declined at years 4 and 5, this is not unusual in long-term follow-up studies [38]. Baseline characteristics between patients with and without 5-yr data were similar except that patients with missing 5-yr questionnaires were more likely to be in a higher NCCN risk group and higher T stage. Patients may have been less willing to complete the QoL questionnaires following relapse. However, since T stage and NCCN risk group were balanced between the randomised groups [12] and data completeness up to 5 yr was also similar between groups, missing data are unlikely to have substantially biased the randomised comparisons reported here. QoL instruments used were amended in response to changing understandings of the strengths and weaknesses of different scales and specifically to ensure better capture of symptoms known to be associated with external beam radiotherapy, but poorly captured in UCLA-PCI. This resulted in lower statistical power for symptoms only in EPIC, including faecal incontinence and rectal bleeding, and means that we cannot rule out small but clinically relevant differences; we therefore strongly encourage future research into the long-term PROs of prostate hypofractionation.

## 5 Conclusions

Results of 5-yr follow-up show similar patient-reported bowel, urinary, and sexual outcomes between schedules, and support the use of moderate hypofractionation as the standard of care for men with intermediate-risk prostate cancer undergoing external beam radiotherapy.

  ***Author contributions*:** Joanne S. Haviland had full access to all the data in the study and takes responsibility for the integrity of the data and the accuracy of the data analysis.

  *Study concept and design*: Staffurth, Wilkins, Syndikus, Khoo, Bloomfield, Parker, Logue, Scrase, Birtle, Malik, Panades, Eswar, Graham, Russell, Ferguson, O’Sullivan, Dearnaley, Hall.

*Acquisition of data*: Staffurth, Syndikus, Khoo, Bloomfield, Parker, Logue, Scrase, Birtle, Malik, Panades, Eswar, Graham, Russell, Ferguson, O’Sullivan, Dearnaley.

*Analysis and interpretation of data*: Haviland, Staffurth, Dearnaley, Hall.

*Drafting of the manuscript*: Haviland, Staffurth, Dearnaley, Hall.

*Critical revision of the manuscript for important intellectual content*: Syndikus, Wilkins, Khoo, Bloomfield, Parker, Logue, Scrase, Birtle, Malik, Panades, Eswar, Graham, Russell, Ferguson, O’Sullivan, Dearnaley, Hall, Cruickshank.

*Statistical analysis*: Haviland.

*Obtaining funding*: Hall, Dearnaley.

*Administrative, technical, or material support*: Cruickshank.

*Supervision*: Dearnaley, Hall.

*Other*: None.

  ***Financial disclosures:*** Joanne S. Haviland certifies that all conflicts of interest, including specific financial interests and relationships and affiliations relevant to the subject matter or materials discussed in the manuscript (eg, employment/affiliation, grants or funding, consultancies, honoraria, stock ownership or options, expert testimony, royalties, or patents filed, received, or pending), are the following: None.

  ***Funding/Support and role of the sponsor*:** We acknowledge support of 10.13039/501100000289Cancer Research UK (C8262/A7253,C1491/A9895,C1491/A15955,C1491/A25351,SP2312/021), the 10.13039/501100000272National Institute for Health Research (NIHR) Cancer Research Network, and NHS funding to the NIHR Biomedical Research Centre at the Royal Marsden NHS Foundation Trust and The Institute of Cancer Research, London.

  ***Acknowledgements*:**We thank the patients and all investigators and research support staff at the participating centres, past and present management group members and the Independent Data Monitoring Committee (Matthew Sydes [chair], Christopher Tyrell, Peter Barrett-Lee, and, previously, Peter Hoskin and Christopher Nutting), and the Trial Steering Committee (Anthony Zietman [chair], Soren Bentzen, Vivian Cosgrove, and Heather Payne) for overseeing the trial.
