# Supplementary material for: Impact of Hypofractionated Radiotherapy on Patient-reported Outcomes in Prostate Cancer: Results up to 5 yr in the CHHiP trial (CRUK/06/016)
Source: Eur Urol Oncol. 2021 Dec;4(6):980–92. doi: 10.1016/j.euo.2021.07.005 (PMC8674146; doi:10.1016/j.euo.2021.07.005)
Supplement: Supplementary file 1 [file mmc1.docx]

**Supplementary Figure 1: Kaplan-Meier plots of time to small or worse bowel, urinary and sexual bother**

| (a) Bowel bother  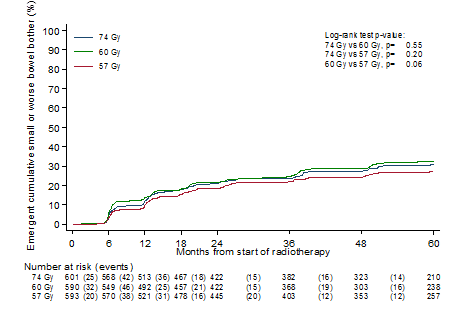 |
| --- |
| (b) Urinary bother  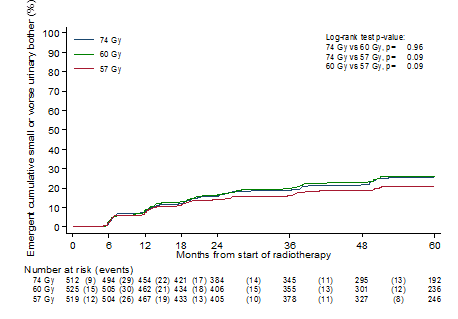 |
| (c) Sexual bother  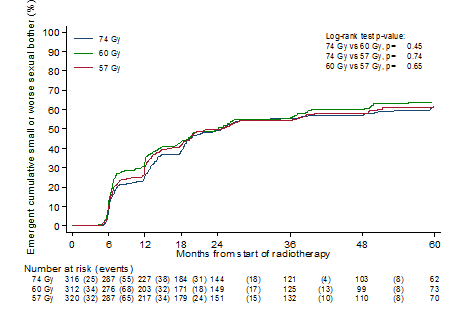 |
