# Supplementary material for: Impact of Hypofractionated Radiotherapy on Patient-reported Outcomes in Prostate Cancer: Results up to 5 yr in the CHHiP trial (CRUK/06/016)
Source: Eur Urol Oncol. 2021 Dec;4(6):980–92. doi: 10.1016/j.euo.2021.07.005 (PMC8674146; doi:10.1016/j.euo.2021.07.005)
Supplement: Supplementary file 2 [file mmc2.docx]

**Supplementary Table 1: Baseline characteristics of patients with and without 5-year questionnaire data**

| **Characteristic** | **5-year questionnaire data available** | | **p-value^1^** |
| --- | --- | --- | --- |
|  | **Yes (%) N=1141** | **No (%) N=959** |  |
| **Age in years;** median (IQR) | 68.4 (63.9, 72.4) | 68.7 (63.9, 73.2) | 0.370^2^ |
| **T-stage** |  |  | <0.001^3^ |
| T1a/b/c/x | 457 (40.0) | 313 (32.6) |  |
| T2a/b/c/x | 591 (51.8) | 543 (56.6) |  |
| T3a/b/x | 93 (8.1) | 101 (10.5) |  |
| Unknown | 0 | 2 (0.2) |  |
| **Gleason score** |  |  | 0.083^3^ |
| <6 | 424 (37.2) | 324 (33.8) |  |
| 7 | 682 (59.8) | 599 (62.5) |  |
| 8 | 35 (3.1) | 36 (3.7) |  |
| **PSA** |  |  | 0.868^3^ |
| 0-4.99 | 77 (6.7) | 69 (7.2) |  |
| 5-9.99 | 444 (38.9) | 363 (37.8) |  |
| 10-19.99 | 529 (46.4) | 457 (47.6) |  |
| >20 | 90 (7.9) | 69 (7.2) |  |
| Unknown | 1 (0.1) | 1 (0.1) |  |
|  |  |  |  |
| *Median (IQR)* | *10.6 (7.3, 15)* | *10.6 (7.4, 14.9)* | 0.829^2^ |
| **NCCN risk group** |  |  | 0.003^3^ |
| Low | 199 (17.4) | 134 (14.0) |  |
| Intermediate | 825 (72.3) | 694 (72.4) |  |
| High | 117 (10.2) | 131 (13.7) |  |
| **Diabetes** |  |  | 0.028^4^ |
| Yes | 1026 (89.9) | 837 (87.3) |  |
| No | 105 (9.2) | 117 (12.2) |  |
| Unknown | 10 (0.9) | 5 (0.5) |  |
| **Hypertension** |  |  | 0.792^4^ |
| Yes | 683 (59.9) | 580 (60.5) |  |
| No | 451 (39.5) | 374 (39.0) |  |
| Unknown | 7 (0.6) | 5 (0.5) |  |
| **Inflammatory bowel or diverticular disease** |  |  | 0.973^4^ |
| Yes | 1093 (95.8) | 918 (95.7) |  |
| No | 42 (3.7) | 35 (3.6) |  |
| Unknown | 6 (0.5) | 6 (0.6) |  |
| **Previous pelvic surgery** |  |  | 0.600^4^ |
| Yes | 1042 (91.3) | 884 (92.2) |  |
| No | 90 (7.9) | 70 (7.3) |  |
| Unknown | 9 (0.8) | 5 (0.5) |  |
| **Symptomatic haemorrhoids in past year** |  |  | 0.903^4^ |
| Yes | 1034 (90.6) | 869 (90.6) |  |
| No | 79 (6.9) | 65 (6.8) |  |
| Unknown | 28 (2.4) | 25 (2.6) |  |
| **Any previous TURP** |  |  | 0.309^4^ |
| Yes | 1013 (88.8) | 869 (90.6) |  |
| No | 104 (9.1) | 76 (7.9) |  |
| Unknown | 24 (2.1) | 14 (1.5) |  |

^1^ Unknown categories excluded from significance tests; ^2^ Wilcoxon rank-sum test; ^3^ Chi-squared test for trend; ^4^ Chi-squared test

**Supplementary Table 2: Bowel, urinary and sexual domain scores at 5 years for UCLA-PCI and EPIC^1^ QoL instruments**

| **Domain score** | **5 years** | | | **60Gy vs. 74Gy** | **57Gy vs. 74Gy** | **60Gy vs 57Gy** |
| --- | --- | --- | --- | --- | --- | --- |
| **N**  **Median (IQR)** | **74Gy/37f** | **60Gy/20f** | **57Gy/19f** | **p-value^2^** | **p-value^2^** | **p-value^2^** |
| **Bowel** | | | | | | |
| **Bowel function;  UCLA-PCI** | N=258  93.7  (79.2-100) | N=282  93.7  (79.2-100) | N=294  93.7  (79.2-100) | 0.721 | 0.750 | 0.964 |
| **Bowel bother; UCLA-PCI** | N=267  100  (75.0-100) | N=285  100  (75.0-100) | N=300  100  (75.0-100) | 0.205 | 0.264 | 0.339 |
| **Bowel function; EPIC** | N=80  92.9  (87.5-98.2) | N=95  96.4  (89.3-100) | N=88  96.4  (85.7-100) | 0.364 | 0.951 | 0.337 |
| **Bowel bother; EPIC** | N=81  95.8  (87.5-100) | N=90  95.8  (83.3-100) | N=90  95.4  (83.3-100) | 0.972 | 0.422 | 0.407 |
| **Bowel summary; EPIC** | N=80  93.2  (88.5-98.1) | N=89  94.2  (86.5-100) | N=85  92.3  (84.6-98.1) | 0.777 | 0.559 | 0.433 |
| **Urinary** | | | | | | |
| **Urinary function;  UCLA-PCI** | N=258  100  (78.0-100) | N=283  100  (78.0-100) | N=293  100  (78.0-100) | 0.791 | 0.865 | 0.973 |
| **Urinary bother;  UCLA-PCI** | N=258  100  (75.0-100) | N=283  100  (75.0-100) | N=293  100  (75.0-100) | 0.748 | 0.487 | 0.721 |
| **Urinary function; EPIC** | N=83  100  (88.4-100) | N=97  100  (91.7-100) | N=96  100  (88.4-100) | 0.310 | 0.831 | 0.392 |
| **Urinary bother; EPIC** | N=76  85.7  (75.0-92.9) | N=89  89.3  (78.6-96.4) | N=84  89.3  (73.2-92.9) | 0.145 | 0.628 | 0.328 |
| **Urinary incontinence; EPIC** | N=76  100  (81.2-100) | N=89  100  (85.5-100) | N=84  100  (79.2-100) | 0.238 | 0.641 | 0.496 |
| **Urinary irritative/ obstructive; EPIC** | N=75  89.3  (82.1-92.9) | N=88  92.9  (82.1-96.4) | N=84  89.3  (78.6-94.6) | 0.177 | 0.939 | 0.189 |
| **Urinary summary; EPIC** | N=76  91.0  (80.6-95.8) | N=88  92.4  (82.3-97.9) | N=84  91.7  (81.2-95.8) | 0.143 | 0.602 | 0.351 |
| **Sexual** | | | | | | |
| **Sexual function;  UCLA-PCI** | N=258  16.6  (3.1-43.7) | N=273  22.9  (3.1-53.1) | N=290  19.7  (4.1-46.9) | 0.071 | 0.188 | 0.558 |
| **Sexual bother; UCLA-PCI** | N=257  50.0  (0-75.0) | N=272  50.0  (12.5-100) | N=286  50.0  (0-100) | 0.019 | 0.048 | 0.754 |
| **Sexual function; EPIC** | N=78  10.0  (0-43.4) | N=94  18.4  (0-48.4) | N=92  26.6  (0-59.2) | 0.399 | 0.162 | 0.465 |
| **Sexual bother; EPIC** | N=76  75.0  (37.5-100) | N=92  50.0  (25.0-100) | N=91  50.0  (25.0-100) | 0.657 | 0.719 | 0.985 |
| **Sexual summary; EPIC** | N=75  18.0  (12.5-48.7) | N=92  24.3  (16.7-48.7 | N=90  30.5  (16.7-62.5) | 0.604 | 0.250 | 0.368 |

All scores range from 0-100, with higher scores representing better quality of life; IQR = interquartile range; ^1^ EPIC-50 used for bowel and urinary domains and EPIC-26 for sexual domains; ^2^ Mann-Whitney test

**Supplementary Table 3: Decline in bowel, urinary and sexual domain scores from baseline to 5 years according to recommended cut-offs for minimal important difference (MID)**

| **Domain score** | **MID^1^** | **Number of patients with decline in domain score >MID from baseline to 5 years / total (%)** | | |
| --- | --- | --- | --- | --- |
|  |  | **74Gy/37f** | **60Gy/20f** | **57Gy/19f** |
| **Bowel function; UCLA-PCI** | 7 | 70/226 (31.0) | 60/224 (26.8) | 60/248 (24.2) |
| **Bowel summary; EPIC** | 15 | 3/51 (5.9) | 8/51 (15.7) | 12/53 (22.6) |
| **Urinary function; UCLA-PCI** | 8 | 56/227 (24.7) | 58/229 (25.3) | 67/246 (27.2) |
| **Urinary summary; EPIC** | 17 | 5/47 (10.6) | 3/49 (6.1) | 6/51 (11.8) |
| **Sexual function; UCLA-PCI** | 8 | 56/104 (53.8) | 69/102 (67.6) | 65/110 (59.1) |
| **Sexual summary; EPIC** | 19 | 9/14 (64.3) | 5/13 (38.5) | 8/18 (44.4) |

^1^ Published in Jayadeppa et al 2012

**Supplementary Table 4: Survival analysis of bowel problems up to 5 years**

| **Bowel endpoints** | **Schedule** | **Small or worse events** | | | | **Moderate or worse events** | | | |
| --- | --- | --- | --- | --- | --- | --- | --- | --- | --- |
|  |  | **Emergent events up to 5 years / N** | **Estimate of 5-year cumulative incidence^1^, % (99%CI)** | **HR (99%CI), p-value for comparison with 74Gy** | **HR (99%CI),**  **p-value for**  **60Gy vs 57Gy** | **Emergent events up to 5 years / N** | **Estimate of 5-year cumulative incidence^1^, % (99%CI)** | **HR (99%CI), p-value for comparison with 74Gy** | **HR (99%CI),**  **p-value for**  **60Gy vs 57Gy** |
| **Overall bowel bother (UCLA-PCI & EPIC)** | **74Gy**  **60Gy**  **57Gy** | 177/601  185/590  162/593 | 39.2 (31.1-48.7)  39.5 (32.4-47.5)  35.4 (28.1-44.0) | 1  1.06 (0.81-1.39), p=0.546  0.87 (0.66-1.15), p=0.200 | 1.22 (0.93-1.61), p=0.062 | 99/631  102/630  91/633 | 20.7 (15.2-28.0)  19.6 (15.1-25.4)  18.3 (13.0-25.3) | 1  1.02 (0.71-1.47), p=0.888  0.88 (0.60-1.27), p=0.365 | 1.17 (0.80-1.69), p=0.281 |
| **Rectal urgency (UCLA-PCI & EPIC)** | **74Gy**  **60Gy**  **57Gy** | 175/566  192/578  165/563 | 37.3 (30.3-45.3)  41.3 (34.1-49.4)  34.7 (28.6-41.7) | 1  1.08 (0.82-1.41), p=0.490  0.91 (0.69-1.20), p=0.395 | 1.19 (0.90-1.56), p=0.104 | 140/590  144/596  125/594 | 30.9 (24.1-39.1)  31.2 (24.1-39.8)  25.1 (19.9-31.2) | 1  1.01 (0.74-1.37), p=0.963  0.85 (0.62-1.17), p=0.202 | 1.19 (0.87-1.62), p=0.162 |
| **Faecal incontinence (EPIC)** | **74Gy**  **60Gy**  **57Gy** | 14/206  26/210  25/224 | 9.7 (4.3-21.0)  14.6 (8.9-23.6)  13.3 (8.1-21.4) | 1  1.88 (0.80-4.41), p=0.053  1.70 (0.72-4.03), p=0.107 | 1.10 (0.53-2.25), p=0.743 | 3/208  17/210  13/225 | 1.8 (0.4-8.0)  9.3 (5.0-16.7)  6.7 (3.3-13.2) | 1  5.75 (1.15-28.88), p=0.002  4.17 (0.80-21.70), p=0.015 | 1.38 (0.53-3.56), p=0.381 |
| **Rectal bleeding (EPIC)** | **74Gy**  **60Gy**  **57Gy** | 9/206  19/211  14/224 | 5.0 (2.1-11.4)  10.0 (5.6-17.5)  9.0 (3.8-20.4) | 1  2.13 (0.75-6.03), p=0.055  1.46 (0.49-4.38), p=0.390 | 1.47 (0.59-3.63), p=0.274 | 3/208  9/211  6/226 | 2.2 (0.4-10.8)  4.8 (2.0-11.1)  4.6 (1.1-17.9) | 1  3.00 (0.54-16.74), p=0.082  1.83 (0.29-11.30), p=0.409 | 1.64 (0.42-6.37), p=0.346 |
| **Loose or liquid stools (UCLA-PCI & EPIC)** | **74Gy**  **60Gy**  **57Gy** | 173/558  177/569  181/565 | 39.4 (32.0-47.9)  38.2 (31.0-46.4)  38.1 (31.6-45.4) | 1  1.00 (0.76-1.32), p=0.994  1.02 (0.78-1.34), p=0.848 | 0.98 (0.74-1.28), p=0.831 | 71/622  87/633  81/632 | 16.0 (11.1-22.7)  16.7 (11.7-23.6)  16.0 (11.4-22.2) | 1  1.22 (0.80-1.84), p=0.229  1.10 (0.72-1.68), p=0.563 | 1.10 (0.74-1.64), p=0.523 |
| **Bowel frequency^2^ (EPIC)** | **74Gy**  **60Gy**  **57Gy** | 33/199  47/204  52/218 | 19.2 (12.5-28.8)  29.5 (19.2-43.6)  28.6 (20.4-39.2) | 1  1.41 (0.79-2.53), p=0.130  1.46 (0.82-2.59), p=0.086 | 0.97 (0.58-1.63), p=0.880 | 5/209  9/213  5/227 | 3.1 (0.9-10.4)  7.8 (2.4-23.7)  2.6 (0.8-8.2) | 1  1.76 (0.42-7.40), p=0.307  0.88 (0.17-4.50), p=0.864 | 1.99 (0.47-8.39), p=0.208 |
| **Crampy pain in abdomen/ pelvis (UCLA-PCI & EPIC)** | **74Gy**  **60Gy**  **57Gy** | 91/620  88/602  108/615 | 19.4 (13.3-27.9)  16.8 (12.8-22.0)  20.9 (16.1-26.9) | 1  0.99 (0.67-1.45), p=0.928  1.19 (0.82-1.71), p=0.229 | 0.83 (0.57-1.20), p=0.199 | 47/636  55/626  59/640 | 10.1 (6.5-15.6)  10.0 (7.0-14.1)  12.1 (8.1-17.9) | 1  1.19 (0.71-1.98), p=0.384  1.23 (0.74-2.03), p=0.309 | 0.97 (0.60-1.57), p=0.861 |
| **Bowel distress (UCLA-PCI)** | **74Gy**  **60Gy**  **57Gy** | 186/416  187/402  163/404 | 52.5 (43.4-62.3)  53.2 (44.5-62.4)  47.8 (39.4-57.0) | 1  1.05 (0.80-1.36), p=0.667  0.84 (0.64-1.11), p=0.107 | 1.24 (0.94-1.63), p=0.044 | 80/503  78/492  61/502 | 19.4 (14.5-25.6)  18.7 (13.9-24.7)  15.3 (10.7-21.7) | 1  0.96 (0.63-1.44), p=0.787  0.70 (0.45-1.09), p=0.041 | 1.36 (0.88-2.12), p=0.069 |

^1^ Estimated at 5 years and 3 months, to allow for late visits; ^2^ Frequency of bowel movements: “small or worse” defined as 3+ per day, and “moderate or worse” 5+ per day.

**Supplementary Table 5: Survival analysis of urinary and sexual problems up to 5 years**

| **Endpoints** | **Schedule** | **Small or worse events** | | | | **Moderate or worse events** | | | |
| --- | --- | --- | --- | --- | --- | --- | --- | --- | --- |
|  |  | **Emergent events up to 5 years / N** | **Estimate of 5-year cumulative incidence^1^, % (99%CI)** | **HR (99%CI), p-value for comparison with 74Gy** | **HR (99%CI),**  **p-value for**  **60Gy vs 57Gy** | **Emergent events up to 5 years / N** | **Estimate of 5-year cumulative incidence^1^, % (99%CI)** | **HR (99%CI), p-value for comparison with 74Gy** | **HR (99%CI),**  **p-value for**  **60Gy vs 57Gy** |
| **Urinary endpoints** | | | | | | | | | |
| **Overall urinary bother (UCLA-PCI & EPIC)** | **74Gy**  **60Gy**  **57Gy** | 126/512  132/525  108/519 | 32.8 (25.3-41.7)  32.6 (25.2-41.5)  27.3 (20.6-35.6) | 1  0.99 (0.72-1.37), p=0.953  0.80 (0.57-1.12), p=0.085 | 1.24 (0.89-1.74), p=0.092 | 78/602  78/599  72/607 | 16.4 (12.2-21.9)  18.2 (12.8-25.4)  18.2 (12.3-26.6) | 1  0.99 (0.65-1.49), p=0.917  0.87 (0.57-1.33), p=0.408 | 1.13 (0.74-1.73), p=0.441 |
| **Loss of urinary control (UCLA-PCI & EPIC)** | **74Gy**  **60Gy**  **57Gy** | 184/438  195/462  171/451 | 52.4 (42.7-62.9)  48.8 (41.8-56.4)  46.3 (38.2-55.2) | 1  0.97 (0.75-1.27), p=0.785  0.84 (0.64-1.11), p=0.109 | 1.15 (0.88-1.51), p=0.177 | 44/644  66/638  44/644 | 9.1 (5.7-14.4)  14.4 (9.6-21.2)  10.0 (6.1-16.1) | 1  1.50 (0.91-2.47), p=0.035  0.95 (0.55-1.65), p=0.806 | 1.58 (0.96-2.61), p=0.017 |
| **Use of urinary pads^2^ (UCLA-PCI & EPIC)** | **74Gy**  **60Gy**  **57Gy** | 39/637  44/642  34/651 | 10.3 (5.6-18.5)  9.2 (5.5-15.1)  9.1 (5.0-16.2) | 1  1.08 (0.61-1.90), p=0.704  0.80 (0.43-1.46), p=0.323 | 1.36 (0.75-2.44), p=0.179 | 5/650  11/652  8/660 | 1.3 (0.3-4.9)  2.7 (1.1-6.4)  1.5 (0.6-3.7) | 1  2.13 (0.53-8.56), p=0.152  1.50 (0.34-6.52), p=0.465 | 1.42 (0.43-4.69), p=0.451 |
| **Haematuria (EPIC)** | **74Gy**  **60Gy**  **57Gy** | 5/206  8/212  7/227 | 4.6 (1.3-15.7)  3.7 (1.4-9.6)  6.4 (1.8-21.2) | 1  1.49 (0.34-6.48), p=0.504  1.17 (0.26-5.32), p=0.789 | 1.26 (0.33-4.77), p=0.657 | 3/207  7/212  5/228 | 2.0 (0.5-8.7)  3.2 (1.1-9.0)  2.4 (0.6-8.9) | 1  2.10 (0.35-12.50), p=0.263  1.35 (0.20-8.94), p=0.712 | 1.55 (0.34-7.03), p=0.449 |
| **Dysuria (EPIC)** | **74Gy**  **60Gy**  **57Gy** | 13/201  26/210  20/221 | 7.3 (3.6-14.5)  13.0 (7.9-20.9)  11.1 (6.3-19.3) | 1  1.95 (0.81-4.69), p=0.048  1.42 (0.57-3.56), p=0.308 | 1.37 (0.64-2.96), p=0.283 | 10/203  17/210  16/223 | 5.4 (2.4-11.8)  8.5 (4.5-15.7)  9.8 (4.9-19.1) | 1  1.64 (0.59-4.58), p=0.225  1.46 (0.52-4.13), p=0.330 | 1.12 (0.46-2.75), p=0.739 |
| **Sexual endpoints** | | | | | | | | | |
| **Overall sexual bother (UCLA-PCI & EPIC)** | **74Gy**  **60Gy**  **57Gy** | 188/316  196/312  197/320 | 73.3 (60.3-84.8)  67.3 (59.1-75.2)  71.0 (61.1-80.3) | 1  1.08 (0.83-1.40), p=0.450  1.03 (0.80-1.35), p=0.741 | 1.05 (0.81-1.36), p=0.653 | 184/395  179/379  187/387 | 58.1 (46.9-69.7)  52.0 (44.0-60.5)  56.2 (47.0-65.9) | 1  0.98 (0.74-1.28), p=0.830  1.02 (0.78-1.33), p=0.906 | 0.96 (0.73-1.26), p=0.700 |
| **Problem with erection quality (UCLA-PCI & EPIC)** | **74Gy**  **60Gy**  **57Gy** | 136/189  162/206  141/194 | 85.5 (73.2-94.2)  85.4 (75.5-92.8)  79.1 (69.1-87.6) | 1  1.14 (0.84-1.54), p=0.249  1.04 (0.76-1.42), p=0.729 | 1.09 (0.81-1.47), p=0.451 | 123/236  141/259  144/257 | 63.1 (51.4-74.8)  63.5 (52.0-74.9)  62.8 (53.5-72.0) | 1  0.99 (0.72-1.36), p=0.935  1.05 (0.77-1.45), p=0.672 | 0.94 (0.69-1.27), p=0.602 |
| **Problem with erection frequency (UCLA-PCI & EPIC)** | **74Gy**  **60Gy**  **57Gy** | 140/187  146/195  134/183 | 85.1 (74.2-93.1)  81.9 (71.8-90.0)  79.8 (69.5-88.3) | 1  0.93 (0.68-1.26), p=0.484  1.01 (0.74-1.38), p=0.916 | 0.92 (0.67-1.25), p=0.470 | 133/212  150/239  139/229 | 78.2 (65.6-88.7)  68.5 (58.7-77.9)  68.5 (58.1-78.4) | 1  0.95 (0.70-1.29), p=0.660  0.95 (0.70-1.30), p=0.669 | 1.00 (0.74-1.35), p=0.999 |
| **Problem with erection on waking morning/ night (UCLA-PCI)** | **74Gy**  **60Gy**  **57Gy** | 83/90  96/100  93/99 | N/A  96.7 (87.9-99.6)  95.8 (88.2-99.1) | 1  0.93 (0.63-1.37), p=0.600  1.06 (0.72-1.56), p=0.680 | 0.88 (0.60-1.28), p=0.371 | 91/112  100/129  110/138 | N/A  80.0 (68.0-89.7)  87.6 (75.1-95.6) | 1  0.75 (0.51-1.10), p=0.046  0.95 (0.66-1.36), p=0.649 | 0.80 (0.56-1.15), p=0.113 |

^1^ Estimated at 5 years and 3 months, to allow for late visits; ^2^ Use of pads: “small or worse” defined as 1+ per day, and “moderate or worse” 3+ per day; N/A = not available (only 1 patient at risk at 5 years and 3 months)

**Supplementary Table 6: General QoL domain scores at 5 years for SF-12, FACT-P and SF-36 QoL instruments**

| **Domain score** | **5 years** | | | **60Gy vs. 74Gy** | **57Gy vs. 74Gy** | **60Gy vs 57Gy** |
| --- | --- | --- | --- | --- | --- | --- |
| **N**  **Median (IQR)** | **74Gy/37f** | **60Gy/20f** | **57Gy/19f** | **p-value^1^** | **p-value^1^** | **p-value^1^** |
| **SF-12^2^** | | | | | | |
| **Mental health composite** | N=79  83.3  (70.8-91.7) | N=94  83.3  (66.7-91.7) | N=95  79.2  (62.5-91.7) | 0.916 | 0.264 | 0.214 |
| **Physical health composite** | N=83  79.2  (54.2-91.7) | N=97  79.2  (58.3-91.7) | N=96  75.0  (50.0-91.7) | 0.689 | 0.846 | 0.503 |
| **FACT-P** | | | | | | |
| **Physical wellbeing^3^** | N=265  26  (23-27) | N=280  26  (23-27) | N=297  26  (23-27) | 0.807 | 0.925 | 0.921 |
| **Social/family wellbeing^3^** | N=263  24  (20-25.7) | N=281  23.3  (19-26) | N=297  24  (20-26) | 0.827 | 0.869 | 0.912 |
| **Emotional wellbeing^4^** | N=264  22.8  (20-24) | N=281  22  (20-24) | N=298  22  (19-24) | 0.690 | 0.410 | 0.666 |
| **Functional wellbeing^3^** | N=263  24  (21-27) | N=281  25  (20-27) | N=299  24.5  (20-27) | 0.353 | 0.612 | 0.680 |
| **Prostate cancer subscale^5^** | N=264  38  (33-42) | N=285  38  (32.7-43) | N=300  39  (32.9-43) | 0.566 | 0.423 | 0.807 |
| **Trial Outcome Index^6^** | N=269  87  (73-94.4) | N=287  88  (75-95) | N=304  88  (73.5-95.2) | 0.613 | 0.543 | 0.941 |
| **FACT-G total score^7^** | N=265  94  (84-101) | N=281  94  (81-102) | N=299  94  (83-101.2) | 0.783 | 0.925 | 0.830 |
| **FACT-P total score^8^** | N=269  132  (112-142) | N=287  130  (113-143) | N=304  131.2  (114-143) | 0.882 | 0.761 | 0.898 |
| **SF-36^2^** | | | | | | |
| **Physical functioning** | N=265  80  (60-95) | N=283  85  (65-95) | N=296  82.5  (65-95) | 0.173 | 0.707 | 0.257 |
| **Role limitations (physical)** | N=262  25  (0-100) | N=282  25  (0-100) | N=296  25  (0-100) | 0.673 | 0.940 | 0.613 |
| **Role limitations (emotional)** | N=263  100  (0-100) | N=282  100  (0-100) | N=298  100  (0-100) | 0.946 | 0.391 | 0.426 |
| **Vitality** | N=259  62.5  (50-75) | N=282  62.5  (50-75) | N=295  62.5  (43.7-75) | 0.838 | 0.376 | 0.274 |
| **Mental health** | N=258  85  (75-90) | N=282  85  (75-95) | N=295  85  (70-90) | 0.464 | 0.535 | 0.164 |
| **Social functioning** | N=270  100  (75-100) | N=289  100  (75-100) | N=306  100  (75-100) | 0.908 | 0.927 | 0.813 |
| **Bodily pain** | N=262  90  (57.5-100) | N=282  90  (65-100) | N=295  80  (57.5-100) | 0.416 | 0.902 | 0.273 |
| **General health** | N=253  70  (55-80) | N=276  70  (55-85) | N=288  70  (50-80) | 0.431 | 0.605 | 0.182 |

^1^Mann-Whitney test; ^2^SF-12 and SF-36 scores range from 0-100; FACT-P scores range from 0-28^3^ or 0-24^4^ or 0-48^5^ or 0-104^6^ or 0-108^7^ or 0-156^8^; higher scores represent better quality of life; IQR = interquartile range

**Supplementary Table 7: Decline in general HRQoL domain scores from baseline to 5 years according to recommended cut-offs for minimal important difference (MID)**

| **Domain score** | **MID^1^** | **Number of patients with decline in domain score >MID from baseline to 5 years / total (%)** | | |
| --- | --- | --- | --- | --- |
|  |  | **74Gy/37f** | **60Gy/20f** | **57Gy/19f** |
| **FACT-P total score** | 8^2^ | 38/114 (33.3) | 42/107 (39.2) | 40/113 (35.4) |
| **General health (SF-36)** | 8 | 42/98 (42.9) | 33/94 (35.1) | 39/104 (37.5) |
| **Physical functioning (SF-36)** | 7 | 56/112 (50/0) | 46/104 (44.2) | 52/115 (45.2) |
| **Role limitations, physical (SF-36)** | 14 | 58/110 (52.7) | 58/103 (56.3) | 63/113 (55.7) |
| **Mental health (SF-36)** | 6 | 22/109 (20.2) | 29/105 (27.6) | 26/109 (23.8) |
| **Role limitations, emotional (SF-36)** | 12 | 34/111 (30.6) | 32/102 (31.4) | 37/113 (32.7) |

^1^ Published in Jayadeppa et al 2012 (SF-36); ^2^ MID range 6-10 for FACT-P total score published in Cella et al 2009; midpoint (8) has been used above
